# On the Molecular Origin of the Tissues

**Published:** 1853-01

**Authors:** 


From the Provincial Medical and Surgical Jour.

*On the Molecular Origin of the Tissues.*

Dr. Bennett read a memoir to the Physiological Society of Edinburgh, the object of which was to prove not only that cells were developed from nuclei, as had been previously ascertained, but likewise that these nuclei themselves originated in smaller bodies, viz., molecules: and that these were the origin of the tissues. He also endeavored to indicate the laws which governed their formation, arrangement, and subsequent development. The author showed, by a reference to the observations of Schleiden and Schwann, that the first step in the organization of all tissues was the coalescence of molecules and granules into a cell-germ, which farther derived a cell-wall from the agglomeration of other molecules. At any period in the progress of evolution the onward progress might be checked, when the structure became disintegrated in a manner inverse to its mode of formation. First, the cell-wall became dissolved, then the nucleus, both of which were reduced to molecules, and then to an amorphous fluid. The author likewise mentioned another form of molecules, which he called secondary. These constitute peculiar secretions. The author next alluded to the origin and mode of formation, with the physiological and pathological importance of these three kinds of molecules, and described the investigations of Ascherson and Melsens. He concluded by pointing out the relation of a knowledge of this molecular formation to the study and treatment of disease. He stated, for instance, that in tubercular diseases the molecules of evolution were deficient, from absence of the fatty element in the chyle; and that, in some other diseases, as those of gouty, rheumatic and scorbutic origin, a cure could only be effected by the introduction of such substances into the blood as favored the production of molecules of transformation. The paper was very elaborate, and commanded a large share of attention.

